# Bacterial Outer Membrane Vesicles Promote Lung Inflammatory Responses and Macrophage Activation via Multi-Signaling Pathways

**DOI:** 10.3390/biomedicines11020568

**Published:** 2023-02-15

**Authors:** Sunhyo Ryu, Kareemah Ni, Chenghao Wang, Ayyanar Sivanantham, Jonathan M. Carnino, Hong-Long Ji, Yang Jin

**Affiliations:** 1 Division of Pulmonary and Critical Care Medicine, Department of Medicine, Boston University, Boston, MA 02118, USA; 2 Department of Cellular and Molecular Biology, University of Texas at Tyler Health Science Center, 11937 US Hwy 271, BMR, Lab D-11, Tyler, TX 75708, USA

**Keywords:** OMV, inflammation, macrophage, activation, bacteria

## Abstract

Emerging evidence suggests that Gram-negative bacteria release bacterial outer membrane vesicles (OMVs) and that these play an important role in the pathogenesis of bacterial infection-mediated inflammatory responses and organ damage. Despite the fact that scattered reports have shown that OMVs released from Gram-negative bacteria may function via the TLR2/4-signaling pathway or induce pyroptosis in macrophages, our study reveals a more complex role of OMVs in the development of inflammatory lung responses and macrophage pro-inflammatory activation. We first confirmed that various types of Gram-negative bacteria release similar OMVs which prompt pro-inflammatory activation in both bone marrow-derived macrophages and lung alveolar macrophages. We further demonstrated that mice treated with OMVs via intratracheal instillation developed significant inflammatory lung responses. Using mouse inflammation and autoimmune arrays, we identified multiple altered cytokine/chemokines in both bone marrow-derived macrophages and alveolar macrophages, suggesting that OMVs have a broader spectrum of function compared to LPS. Using TLR4 knock-out cells, we found that OMVs exert more robust effects on activating macrophages compared to LPS. We next examined multiple signaling pathways, including not only cell surface antigens, but also intracellular receptors. Our results confirmed that bacterial OMVs trigger both surface protein-mediated signaling and intracellular signaling pathways, such as the S100-A8 protein-mediated pathway. In summary, our studies confirm that bacterial OMVs strongly induced macrophage pro-inflammatory activation and inflammatory lung responses via multi-signaling pathways. Bacterial OMVs should be viewed as a repertoire of pathogen-associated molecular patterns (PAMPs), exerting more robust effects than Gram-negative bacteria-derived LPS.

## 1. Introduction

Gram-negative bacteria-induced pneumonia has features of serious concern, including being highly efficient at acquiring antibiotic drug resistance [1] and having a significantly higher incidence of bacteremia among adult patients with septic shock and systemic inflammatory response syndrome (SIRS) [2,3]. Furthermore, severe pneumonia-associated lung inflammation is one of the main causes of acute respiratory distress syndrome (ARDS), a devastating clinical entity often seen in critical care units [4]. Macrophages (Mϕs) form the first line of host defense and innate immunity toward microbial pathogens. Upon activation, they release early response cytokines/chemokines which, in turn, mediate the recruitment of neutrophils and exudate Mϕs and lymphocytes to the site of infection, ultimately resulting in the clearance of pathogens [5]. However, these inflammatory lung responses are a “double-edged sword”. The high mortality and morbidity after a bacterial infection are often the result of an imbalance between host bactericidal effects and an excess inflammatory response [6,7]. The mechanisms involved in the development and propagation of lung inflammation and the appropriate return to tissue homeostasis remain incompletely understood and have significantly impeded the development of novel diagnostic and therapeutic agents.

The pathogenesis of lung inflammation and subsequent return to tissue homeostasis is an active, tightly regulated process [5]. Precise and dynamic interactions between the host defense system and microbial pathogens play a key role of innate immunity. Accumulating evidence supports the existence of a novel mechanism of host–microbe interactions via nanosized extracellular vesicles [8]. Almost all types of cells release extracellular vesicles (EVs), including bacteria [9]. The vesicles released from the outer membranes of Gram-negative bacteria are referred to as bacterial outer membrane vesicles (OMVs) [10]. Similar to EVs, OMVs are believed to facilitate communication among themselves, with other microorganisms in their environment, and with the host [11]. These vesicles are potentially involved in the trafficking of bacterial cell signaling biochemicals, which may include DNA, RNA, proteins, endotoxins, and virulence molecules [11]. OMVs are believed to be strong immuno-modulators and may be utilized as potent pathogen-free vaccines [12].

Germline-encoded pattern-recognition receptors (PRRs) are responsible for the initial detection of microbial pathogens, such as bacteria, viruses, and fungi. The pathogen-specific molecular signatures often recognized by PRRs are named pathogen-associated molecular patterns (PAMPs). PRR–DAMPs/PAMPs interaction leads to the activation of downstream signaling pathways, resulting in the activation of innate immune responses and inflammation [13]. These processes are crucial for the clearance of the invading microbial pathogens; however, they also potentially induce organ damage and failure by promoting overwhelming inflammation [14]. In human cells, Toll-like receptors (TLRs), RIG-I-like receptors (RLRs), Nod-like receptors (NLRs), AIM2-like receptors (ALRs), C-type lectin receptors (CLRs), and intracellular DNA sensors such as cGAS are all distinct PRRs [15]. The TLR family is the best characterized PRR; in it, 13 members (TLR1–TLR13) have been identified in mouse. TLRs recognize PAMPs and DAMPs, recruit Toll/IL-1 receptor (TIR) domain-containing adaptor proteins such as MyD88 and TRIF, and subsequently initiate signal transduction pathways to regulate the generation and secretion of cytokines, chemokines, and type I IFNs [15]. Based on their cellular locations, TLRs are divided into two major subfamilies: cell surface TLRs and intracellular TLRs [15]. TLR1, TLR2, TLR4, TLR5, TLR6, and TLR10 belong to the former, while TLR3, TLR7, TLR8, TLR9, TLR11, TLR12, and TLR13 are intracellular ones mainly locating in the endosome and cytosol [15].

Among all TLRs, cell surface TLR4 is known to recognize lipopolysaccharide (LPS), which is the main endo-toxicant derived from Gram-negative bacteria [15]. TLR1, 2, and 6 recognize various types of PAMPs, including lipoproteins, peptidoglycans, and lipotechoic acids [15]. Bacterial flagellin stimulates surface TLR5-dependent host defense [15]. Intracellular TLRs mainly recognize nucleic acids derived from microbial pathogens or diseased host cells [15]. Among intracellular TLRs, TLR3 is known to recognize viral double-stranded RNA (dsRNA) [15], while TLR7 recognizes single-stranded RNA (ssRNA) from viruses [15]. TLR9 recognizes CpG-DNA motifs in bacterial and viral DNA [15]. Previous reports suggest that OMVs derived from *Acinetobacter baumannii* (*A. baumannii*), a Gram-negative bacterium associated with hospital-acquired and ventilator-related pneumonia, increase the release of chemokines and cytokines in mouse lungs [16]. This effect is likely via both TLR2- and TLR4-mediated pathways. A few recent reports further showed that macrophages exposed to Gram-negative bacterial OMVs undergo mitochondrial apoptosis, pyroptosis, and NLRP3 inflammasome activation [17,18,19,20].

In recent years, we have explored the detailed functions and molecular mechanisms of OMVs in the pathogenesis of sepsis, SIRS, and ARDS. Our present report, in addition to agreeing with the previously reported conclusions, identifies several novel findings as detailed in our results and discussion. Our proposed work uncovers potentially novel therapeutic/diagnostic targets and strategies for treating Gram-negative bacterial pneumonia-induced inflammation/injury, which is highly relevant to clinical practice. Furthermore, our study illustrates a potentially novel mechanism by which pathogens interact with living host cells and regulate damage-associated molecular patterns (DAMPs) and pathogen-associated molecular patterns (PAMPs). Further significant aspects include a broader elucidation of the molecular mechanism linking OMVs with well recognized innate immune and inflammatory pathways. To date, the mechanisms by which bacterial OMVs regulate Mϕ activation and lung inflammation have not been completely revealed. In this report, we extrapolate all the above observations to the other TLRs by examining the signaling of TLR2 and TLR4. Furthermore, we identify several specific PAMPs and DAMPs which are responsible for TLR activation. We also show that Gram-negative bacterial OMVs induce macrophage pro-inflammatory activation via multiple layers of signaling in a time-dependent manner, suggesting that OMVs carry more potent and broad cellular effects compared to LPS. By identifying novel cellular targets, our report provides several potentially novel markers for the development of diagnostic tools and therapeutic strategies.

## 2. Materials and Methods

### 2.1. Cells, Cell Culture, Reagents, and Supplies

Dulbecco’s Modified Eagle’s Medium (DMEM), RPMI-1640, phosphate-buffered saline (PBS). Fetal bovine serum (FBS), and Penicillin Streptomycin (Pen Strep) were purchased from Gibco™, Thermofisher, Waltham, MA, USA. Bone marrow derived macrophages (BMDMs) were isolated from wild type (WT) C57BL/6 mice (male and female) between 6 to 8 weeks of age (Jackson Laboratory, Bar Harbor, ME, USA) as previously described [21]. DMEM complete media with 30% L929 conditional media were used to culture BMDM for 7 days until fully differentiated before performing any further experiments or assays. Cultured BMDMs were incubated with OMVs, BALF exosomes (EXOs), or lipopolysaccharide (LPS) (Sigma-Aldrich, St. Louis, MO, USA) with the desired time and amount based on the experimental design. MHS cells (ATCC, Manassas, VA, USA) were cultured with RPMI-1640 with 10% FBS and 1% Pen Strep. All cells were maintained in 37 °C incubator (Fisher Scientific, Hampton, NH, USA) with 5% CO2 95% air.

### 2.2. Mice

Wild type (WT) C57BL/6 mice (male and female), 8 weeks of age, and TLR4 knockout mice, B6.B10ScN-Tlr4lps-del/JthJ, 8 weeks of age, were obtained from the Jackson Laboratory (Bar Harbor, ME, USA). All protocols and methods involving animals were approved by the Institutional Animal Care and Use Committee of Boston University in accordance with approved guidelines.

### 2.3. Animal Study

Outer-membrane vesicles (OMV) were intratracheally instilled into the mouse lung (ug of OMVs in total protein). At 24 h after administration, BALFs were collected from the mouse lungs. Lung tissues that were not subjected to the BALF collection were used for the histology and isolation of lung tissue inflammatory cells.

### 2.4. BALF and Inflammatory Cell Counts

To induce lung inflammation, WT mice were anaesthetized and intratracheally instilled with the amounts of OMVs indicated below. Twenty-four hours after the instillation, mice were euthanized by aerosolized isoflurane overdose and their BALF was collected. For cytospin preparations, the cell suspensions were cytocentrifuged at 300× *g* for 5 min using a Shandon Cytospin 4 (Thermo Fisher Scientific, Rockford, IL, USA). Slides were air-dried and stained with PROTOCOL Hema 3 fixative and solutions (Fisher Scientific, Hampton, NH, USA). Differential cell counts were evaluated under a microscope.

### 2.5. Isolation of Lung Infiltrating Cells in Mice and Cell Counts

Briefly, the left lung lobes were collected, washed with sterile 1x PBS, and placed in Petri dishes with RPMI 1640 medium (2 mL). Tissues were minced with forceps and scissors to 1 mm size chunks. Once the lung had been dissected, the tissue was incubated with 3 mL of digestion medium containing dispase II (Roche, Indianapolis, IN, USA) and Collagenase A (Roche, Indianapolis, IN, USA) at 37 °C under agitation (250 rpm) conditions for 60 min. Cells were dispersed with a 10 mL syringe fitted with an 18-gauge needle (10 times). Cells were filtered using a cell strainer (100 μm) and then centrifuged at 4 °C, 300× *g* for 5 min. After removing the supernatant by aspirating, 1 mL of Red Blood cells lysing buffer (RBC lysis buffer) (Thermofisher, Waltham, MA, USA) was added and incubated at room temperature for 1 min.

After RBC lysis, the cells were centrifuged and resuspended in PBS. Differential cell counts were evaluated using cytospin preparations, as described above.

### 2.6. Histopathologic Evaluation

Lungs were excised after whole body perfusion, immediately fixed with 4% paraformaldehyde (Fisher Scientific, Hampton, NH, USA), sectioned (5 μm), stained with hematoxylin and eosin (Sigma, St Louis, MO, USA), and visualized using a microscope.

### 2.7. Outer-Membrane Vesicles (OMV) and Exosome (EXO) Isolation and Characterization

*E. coli* O6:K2:H1 (ATCC, Manassas, VA, USA) were cultured overnight in Luria-Bertani medium at 37  °C rotator with speed 180 rpm until the desired OD_600_ was reached. The cultured *E. coli* were then pelleted and filtered as previously described [22], followed by OMV purification using ExoBacteria OMV Isolation Kits (SBI, Palo Alto, CA, USA), based on the manufacturer’s protocol. The protein content in the isolated OMVs was measured using Bradford assays with Coomassie Protein Assay Reagent (Thermo Fisher Scientific, Waltham, MA, USA). Exosomes were isolated from the bronchoalveolar lavage fluid (BALF) of WT mice. To obtain BALF, a total of 2 mL of PBS was used for the collection, as previously described [23]. BALF was centrifuged at 300× *g* for 10 min to remove floating cells, followed by 2000× *g* for 20 min to separate apoptotic bodies, and 16,000× *g* for 40 min to separate microvesicles [24]. The resulting supernatants were then ultracentrifuged at 100,000× *g* for 1 h to obtain exosomes [24]. TEM images of OMVs were generated at the Experimental Pathology Laboratory Core, Boston University School of Medicine.

### 2.8. ELISA and Array Analyses

To quantify the cytokine and chemokine levels in BALF and cells, ELISA Kits (R&D Systems, Minneapolis, MN, USA) were used according to the manufacturer’s protocol. The expression of toll-like receptors, chemokines, interleukins, and other inflammation-related genes in OMV-treated BMDMs was measured using an RT² Profiler PCR Array for Mouse Inflammatory Response and Autoimmunity (QIAGEN, Valencia, CA, USA). To check chemokine expression in MHS cells treated with exosomes or OMVs, a Proteome Profiler^TM^ Antibody Arrays–Mouse Chemokine Array (R&D Systems, Minneapolis, MN, USA) was used. Inflammatory responses from BMDMs were checked using Acute Inflammation Response M96 (BioRad, Hercules, CA, USA) collection panels. All arrays were performed according to the manufacturer’s protocol.

### 2.9. RNA Isolation, Reverse Transcription, and qRT-PCR

For purification of total RNA from cells, RNeasy Plus Mini Kits (QIAGEN, Valencia, CA) were used. Single stranded cDNAs were prepared using High-Capacity cDNA Reverse Transcription Kit (Thermo Fisher Scientific, Waltham, MA, USA). For real-time quantitative PCR (qPCR), PowerUp^TM^ SYBR^TM^ Green Master Mix (Applied Biosystems, Foster City, CA, USA) and QuantStudio 3 system (Applied Biosystems, Foster City, CA, USA) was used. The list of primers is shown in Table 1.

### 2.10. Macrophage Phagocytosis, ROS, and Migration Assays

Phagocytosis assays were performed using pHrodo™ Red and Green BioParticles^®^ Conjugates for Phagocytosis (Thermo Fisher Scientific, Waltham, MA, USA) in which the pHrodo™ Green *E. coli* BioParticles^®^ Conjugate was used on *E. coli* OMV-treated BMDMs. The procedure and analysis were performed according to the manufacturer’s protocol. ROS levels in OMV-treated cells were measured using DCFDA Cellular ROS Detection Assay Kit and performed according to the product manual (Abcam, Cambridge, MA, USA). Macrophage migration assays were performed using 6.5 mm diameter inserts with pore size of 8.0 μm (Corning Incorporated, Corning, NY, USA), as previously described [25]. BMDMs (3 × 10^4^) were placed in the inner chamber and 10% FBS was added to the outer well with or without OMVs, followed by 24 h incubation. Then, 4% formaldehyde was used for fixation and hematoxylin was used for staining. A light microscope was used to count migrated cells [25].

### 2.11. Statistics

Non-parametrical statistical analysis was performed by a One-way Analysis of Variance (ANOVA) test with Tukey’s multiple comparisons and two-tailed unpaired Student’s *t*-test to determine significant differences between the experimental groups.

## 3. Results

### 3.1. Gram-Negative Bacterial OMVs Induce Macrophage Pro-Inflammatory Activations

**Figure 1:**
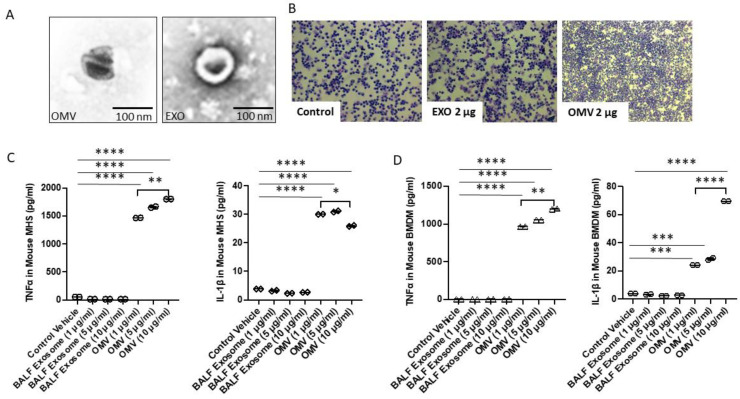
Effects of OMVs on MHS cells. Effects of OMVs on MHS cells. (**A**) TEM images of OMV and EXOs. Size similarity between OMV and EXO is shown. (**B**) Representative images of BALF cells collected from PBS, EXO-treated, and OMV-treated mice. 60 µL PBS, 2 µg of EXO or OMV with 60 µL PBS were instilled intra-tracheally (i.t.) into WT mice. (**C**,**D**) TNF-α and IL-1β levels in cells treated with control (elution buffer), exosomes (control), and OMVs in MHS cells (**C**) or BMDM (**D**). 24 h after stimulation, the above two cytokines were determined using ELISA (*n* = 2). Results were expressed as mean ± SD. Statistical Analysis was performed non-parametrically using the One-way Analysis of variance (ANOVA) with Tukey’s multiple comparison tests to determine significant differences between the experimental groups. * *p* < 0.05, ** *p* < 0.01, *** *p* < 0.005, and **** *p* < 0.001 set as Statistical significance.

We initially performed an in vitro study to explore the effects of Gram-negative bacterial OMVs on macrophage activation. We used PBS to suspend the OMVs. To avoid the non-specific triggering of cell signaling pathways, we initially included host derived EXOs as one of the controls (Figure 1A). Therefore, two controls were used in this study: one was PBS alone and the other was host cell-derived exosomes (EXOs). The term “exosomes” usually refers to the smallest of extracellular vesicles (EVs), ranging from 20 to 200 nm. As shown in Figure 1A, EXOs fall into a similar size range as OMVs. Therefore, EXOs were used as an additional control. Next, to confirm that EXOs and PBS could both serve as a control in vivo, we treated mice with PBS, EXOs (2 mg in 50 µL PBS), and OMVs (2 mg in 50 µL PBS) by intratracheal instillation. We found that only treatment with OMVs induced significant cell infiltration in BALF compared to PBS and EXOs (Figure 1B). The effects of OMVs on macrophages, including systemic circulating macrophages and lung alveolar macrophage, are summarized below.

### 3.2. Gram-Negative Bacterial OMVs Induce Cytokine/Chemokine Release from Both Systemic and Lung Macrophages

**Figure 2:**
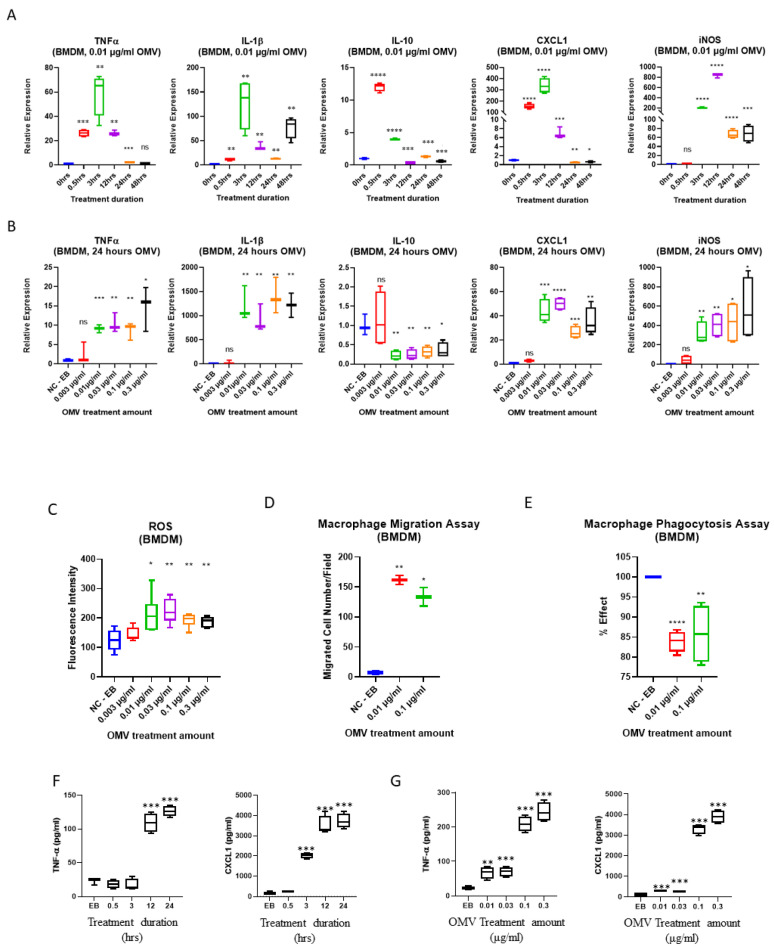
Effects of OMVs on BMDMs. (**A**) Effects of OMVs (0.01 µg/mL) on the cytokine and iNOS production in BMDMs (time course). (**B**) Effects of OMVs (24 h) on the cytokine and iNOS production in BMDMs (dose response). (**A**,**B**) Expression levels of TNFα, IL-1β, IL-10, CXCL1, and iNOS were analyzed using real-time PCR. *n* = 3 or 4. TBP is used as the housekeeping gene. (**C**) Effect of OMVs on ROS production in BMDM. BMDM were treated with OMVs for 3 h with the indicated doses. Data represents *n* = 6. (**D**) Migration assay of OMV-treated BMDM. BMDMs were treated with OMVs (0.01 µg/mL or 0.1 µg/mL respectively). Migration assays were analyzed. (**E**) Effect of OMVs on Phagocytosis in BMDMs. (**D**,**E**) BMDMs were treated with the indicated amounts of OMVs for 24 h (*n* = 4). (**F**–**G**) Time and dose dependent effect of OMVs on the production of proinflammatory mediators in BMDM using ELISA. (**F**) Cells were treated for 0.5 h, 3 h, 12 h or 24 h in in 0.01 µg/mL OMVs and (**G**) Cells were treated for 24 h in different concentrations of OMVs (0.01, 0.03, 0.1, 0.3 µg/mL). The sandwich-ELISA method was used to assess the secretory levels of proinflammatory mediators TNF-α and CXCL-1 in the culture supernatant. Results were expressed as mean ± SD. Statistical Analysis was performed using two-tailed unpaired Student’s *t*-test. Mean  ±  SD is plotted (*n* = 2 or 3). * *p* ≤ 0.005, ** *p* ≤ 0.001, *** *p* ≤ 0.0005, **** *p* ≤ 0.001 versus the control (0 hrs. in A, EB treated in B, C, D); EB—Elution Buffer, hrs.—Hour and ns—non-significant.

Macrophages act as the first line of host defense and innate immunity [26]. We next studied the effects of OMVs on macrophage polarization. As shown in Figure 1C,D, alveolar macrophages (MHS) and bone marrow-derived macrophages (BMDMs) were exposed to a variety of OMVs, and after 24 h, TNF-α and IL-1β were analyzed in the cell supernatant. Compared with host-derived exosomes (the same size as bacterial OMVs), OMVs robustly induced TNF-α and IL-1β secretion in a dose-dependent manner. This observation was confirmed using both MHS macrophage cells and BMDM primary macrophages. We found that as early as 3 h after exposure to OMVs, the majority of cytokines/chemokines were significantly elevated compared to untreated cells. The increased cytokines gradually subsided after 24 h. Among them, IL-10, widely considered an ‘anti-inflammatory’ cytokine, was transiently induced initially at 30 min after exposure to OMVs. However, IL-10 levels rapidly subsided back to baseline after a few of hours. During the first 3 to 24 h, TNF-α, IL-1β, CXCL1, and inducible nitric oxide synthase (iNOS) were all robustly elevated (Figure 2A). Next, with 24 h as the time point, the dose-response from OMV treatment was evaluated. As shown in Figure 2B, a significant dose-response was observed in the secretion of TNF-α, IL-1β, CXCL1, and nitric oxide (NO), but not IL-10, from BMDMs after exposure to OMVs. We further confirmed the above-mentioned results using ELISA (Figure 2F,G). In this experiment, we used BMDMs obtained from female mice to verify our observations. Using TNF-α and CXCL1 as an example, we showed that BMDMs released significantly more cytokines after exposure to OMVs.

### 3.3. Gram-Negative Bacterial OMVs Induce Cell Migration, Phagocytosis, ROS Generation, and NO Production

Next, we evaluated the effects of OMVs on macrophage migration and ROS generation, as well as their phagocytosis capability. As shown in Figure 2C,D, OMVs markedly induced macrophage migration and ROS generation. Interestingly, decreased phagocytosis was noted in BMDMs 24 h after exposure to OMVs, suggesting that complex signaling pathways are involved in the effects of OMVs (Figure 2E).

### 3.4. Gram-Negative Bacterial OMVs Induce Inflammatory Lung Responses In Vivo

**Figure 3:**
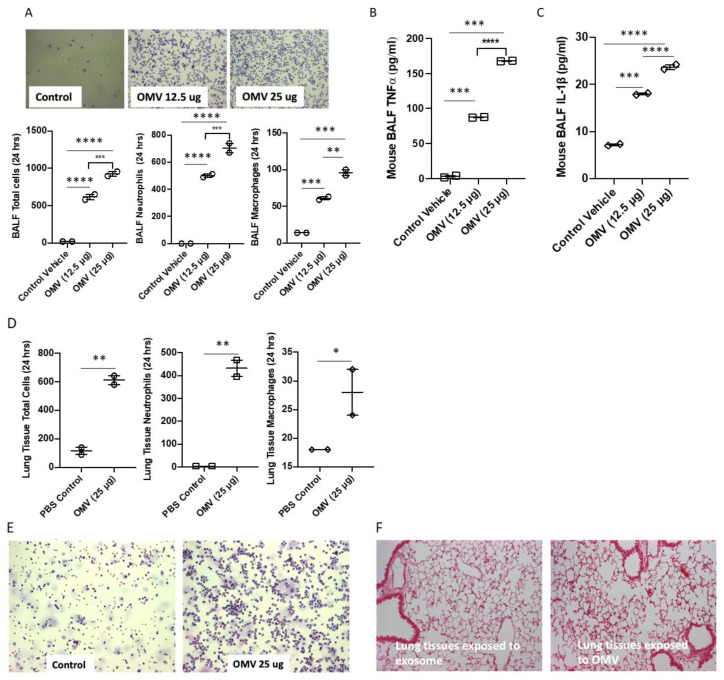
OMVs induced inflammatory lung responses. (**A**) Mice were exposed to different doses of OMVs. After 24 h, BALF were collected, and cells in BALF were stained using H&E staining. The total cell counts (left, lower panel), neutrophil counts (middle, lower panel), and macrophage counts (right, lower panel) were shown. (**B**,**C**) The TNF-α (**B**) and IL-1β (**C**) were determined in BALF using ELISA. (**D**) Effects of OMVs (25 µg/mL) on total cell counts infiltrating the lung tissues (left), on neutrophil counts (middle), and macrophage counts (right). (**E**,**F**) Mice were treated with OMVs (25 µg/mL) i.t., after 24 h, cells infiltrating lung tissue (**E**) were stained using H&E staining; Lung tissue (**F**) was also stained using H&E staining. Results were expressed as mean (*n* = 2 individual experiments) ± SD. Statistical Analysis was performed non-parametrically using the One-way Analysis of variance (ANOVA) with Tukey’s multiple comparison tests to determine significant differences between the experimental groups. * *p* ≤ 0.005 ** *p* ≤ 0.001, *** *p* ≤ 0.0005, **** *p* ≤ 0.001 vs. the control (PBS treated) or 12.5 µg OMV treatment group.

We hypothesized that Gram-negative bacterial OMVs play a role in mediating inflammatory lung responses in the presence of bacterial infections. To determine whether the OMVs shedding from live bacteria play a functional role, we examined the effects of bacterial OMVs on BALF cells. As shown in Figure 3A, C57BL/6 mice were treated with PBS and two different doses of OMVs through intratracheal instillation. After 24 h, BALF total cell counts and BALF macrophage/neutrophil counts were analyzed. We found that BALF total cell counts, neutrophil counts, and macrophage counts all increased robustly (Figure 3A) in the presence of OMVs. Consistently, we found that bacterial OMVs promoted a robust elevation of pro-inflammatory cytokines in BALF, expressed by TNF-α and IL-1β (Figure 3B,C).

In addition to analyzing BALF, we further assessed the effects of OMVs on lung tissue in vivo. As illustrated in Figure 3D, after exposure to bacterial OMVs, interstitial macrophages, interstitial neutrophils, and total infiltrated interstitial cell counts were all elevated in the presence of OMVs. In Figure 3E,F, we show that cell counts in BALF (Figure 3E) and cell infiltration in lung tissue (Figure 3F) were robustly elevated. Increased exudate, cellular infiltration, and alveolar septal thickening was found in lung tissue exposed to bacterial OMVs (right) compared to control (left).

### 3.5. Gram-Negative Bacterial OMVs Induce Multiple Pro-Inflammatory Cytokines and Chemokines in Macrophages In Vitro

**Figure 4:**
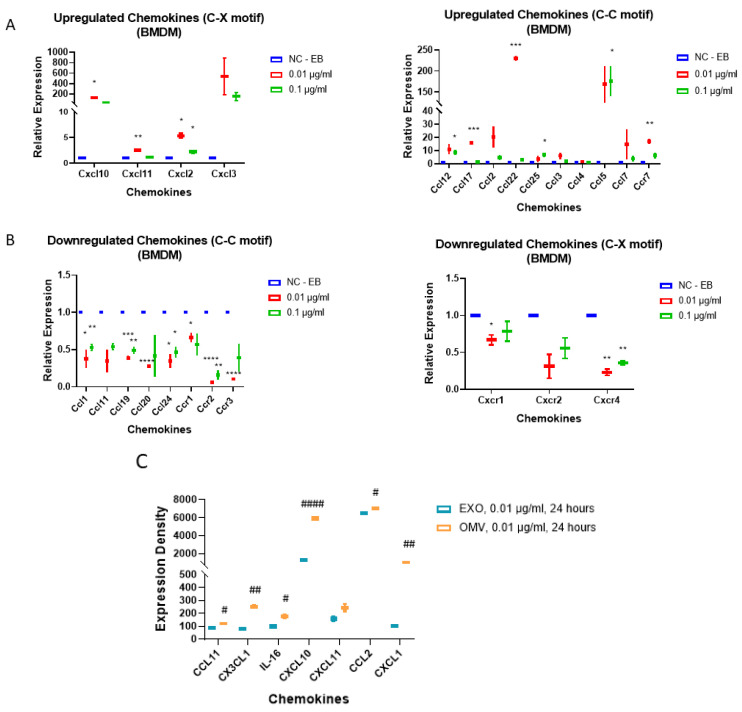
OMVs treatment in macrophages induces pro-inflammatory cytokines and chemokines. (**A**,**B**) BMDM were treated with OMVs at the indicated doses, after 24 h, mouse inflammation and autoimmune arrays were used to screen for candidates involved in OMV-stimulated inflammatory responses. Panel (**A**) shows upregulated chemokines and panel (**B**) shows downregulated chemokines. To visually present the up- and down-regulation resulted from OMV treatment, control groups were normalized to 1. (**C**) A mouse chemokine array was performed to screen for alterations in chemokine levels resulted from equal amounts of EXO or OMV treatment in MHS. Cells were treated with 0.01 µg/mL OMV or EXO for 24 h. (**C**) Representative graph showing 7 of the 25 chemokines profiled by the chemokine array. Data points were analyzed using two-tailed unpaired Student’s *t*-test to compare a treatment group to the control group; Mean  ±  SD; * *p* ≤ 0.005, ** *p* ≤ 0.001, *** *p* ≤ 0.0005, **** *p* ≤ 0.001 versus the control (Negative control EB treated); # *p* ≤ 0.005, ## *p* ≤ 0.001, #### *p* ≤ 0.001 vs. Exosome-treated.

From the above in vivo data, we noted a significant induction of neutrophil infiltration. To investigate whether macrophages release robust amounts of chemokines which potentially drive neutrophil and macrophage migration/infiltration, we next explored the cytokine/chemokine profiles which were altered after OMV treatment in macrophages. Using both BMDMs and MHS cells, we performed cytokine/chemokine arrays after treatment with OMVs. As shown in Figure 4A, upregulated chemokines were observed: C-C motif (panel A left) and C-M motif (panel A right). The downregulated chemokines are shown in Figure 4B. Next, we tested the chemokine expression in EXO-treated MHS cells. In Figure 4C, EXO-treated cells served as a control, while OMV-altered chemokines were the profiles. The major upregulated/downregulated chemokines in the OMV treated MHS cells are displayed in Table 2.

### 3.6. In Addition to TLR4, Gram-Negative Bacterial OMVs Induce Macrophage Activation via Multiple Signaling Pathways

**Figure 5:**
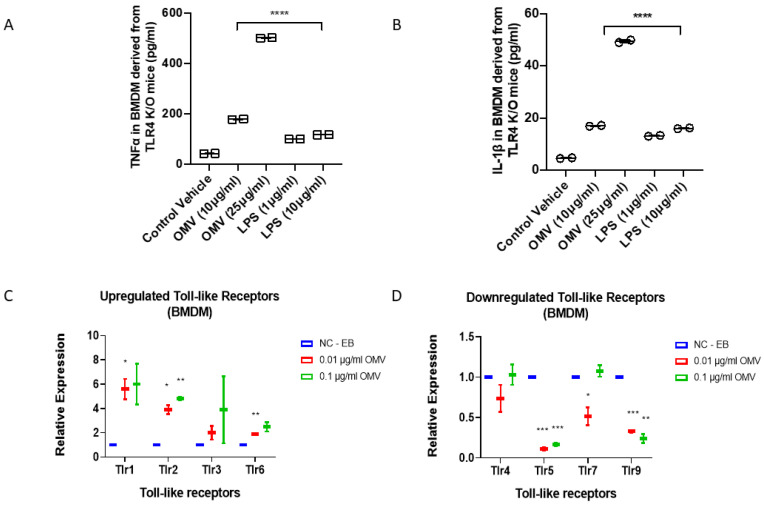
OMVs induce macrophage activation via multi-signaling pathways. (**A**,**B**) BMDMs were obtained from TLR4 knockout mice and then treated with OMVs (10–25 µg/mL) or LPS (1–10 µg/mL). After 24 h, TNF-α (**A**) and IL-1β (**B**) levels in the cell culture supernatant were determined using ELISA. Results are presented using mean  ±  SD from at least two independent experiments (* *p* ≤ 0.01). (**C**,**D**) BMDMs were treated with OMVs in the dose as indicated. After 24 h, mouse inflammation and autoimmune arrays were used to screen for the candidates involved in OMV-stimulated inflammatory responses. The upregulated genes were shown in (**C**) and downregulated genes were shown in (**D**). Control groups were normalized to “1” and folds of increase/decrease were presented for the indicated candidates. Data points were analyzed using two-tailed unpaired Student’s *t*-test to compare with LPS treated or EB treated group; Mean (*n* = 2)  ± SD; * *p* ≤ 0.005 ** *p* ≤ 0.001, *** *p* ≤ 0.0005, **** *p* ≤ 0.001 vs. the control (EB treated) or 10 µg LPS treatment group.

**Figure 6:**
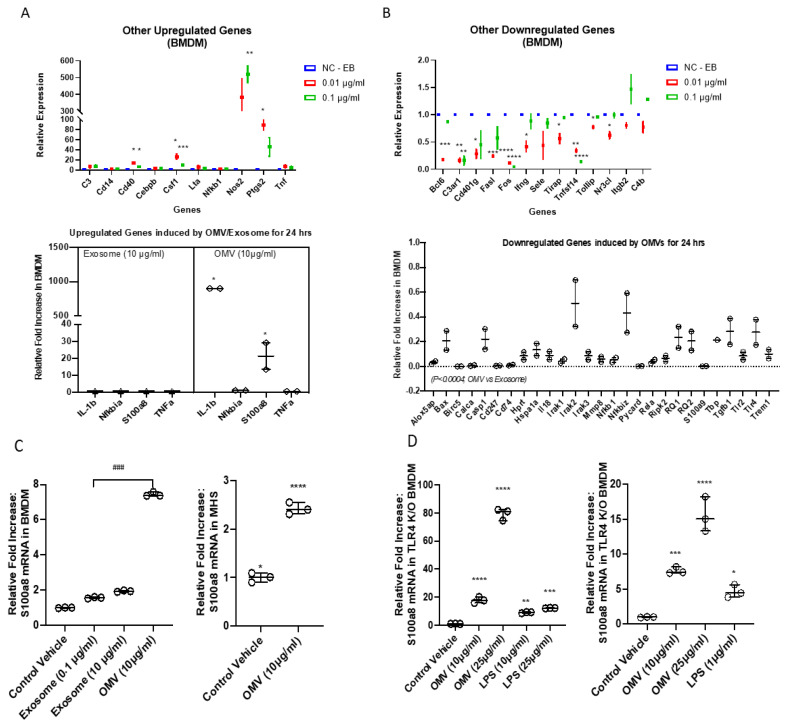
Expression profile of mouse inflammatory genes. (**A**,**B**) The top panel shows results from mouse inflammation and autoimmune array of BMDM treated with the indicated amounts of OMVs for 24 h. In the bottom panel, the profile of mRNA expression of inflammatory target genes was analyzed by M96 PrimePCR array plates utilizing the OMV or exosome-treated samples and plotted against those of their controls. Mouse BMDM were treated with 10 µg/mL OMV or EXO for 24 h. (**A**) A summary of the upregulated genes. (**B**) A summary of the downregulated genes. (**C**,**D**) The validated mRNA expression of S100a8 in BMDM (C-left), MHS (C-right), and TLR4 K/O BMDM (**D**), after treatment with control vehicle, and the indicated concentration of exosome and OMV. Results were expressed as mean (*n* = 2) ± SD. Statistical Analysis was performed non-parametrically using the One-way Analysis of variance (ANOVA) with Tukey’s multiple comparison tests to determine significant differences between the experimental groups. * *p* ≤ 0.005, ** *p* ≤ 0.001, *** *p* ≤ 0.0005, **** *p* ≤ 0.001 versus the control (EB treated); ### *p* ≤ 0.0005 vs. Exosome.

Gram-negative bacteria-derived LPS is known to induce macrophage activation via TLR4-mediated signaling [27]. We next determined whether TLR4 receptors were required for OMVs to activate macrophages. BMDMs were isolated from TLR4 knockout (KO, null) mice as described in Material and Methods. We repeated the cytokine assays using both OMVs and LPS on the TLR4 KO BMDMs. Interestingly, after 24 h, deletion of TLR4 blunted the LPS-induced TNF-α and IL-1β secretion but not the OMV-mediated upregulation (Figure 5A,B). Based on the above observations, we hypothesized that TLR receptors other than TLR4 play a role in mediating the effect of OMVs on macrophages. We therefore analyzed the effects of OMVs on a panel of TLR expression. TLR1 and TLR2 transcription were upregulated after exposure to OMVs in BMDMs, while TLR5 was downregulated. TLR4 was not altered significantly (Figure 5C,D). Furthermore, using a gene expression array, we screened a variety of signaling molecules and identified multiple pathways which might have been altered after OMV exposure, including, but not limited to, nucleotide-binding oligomerization domain-containing protein 2 (*NOD2*) and prostaglandin-endoperoxide synthase 2 (PTGS2) pathways (Figure 6A,B).

### 3.7. Gram-Negative Bacterial OMVs Induce Pro-Inflammatory DAMPs Expression in Macrophages

OMVs seem to have more potent effects on macrophage activation compared to LPS. Using proteomics, we identified numerous proteins and molecules which may function as PAMPs, in addition to LPS. Here, we further explored whether OMVs induce host cell-derived DAMPs. As shown in Figure 6 (panel C), BMDMs and MHS cells were treated with OMVs (10 µg/mL), host EXOs (10 µg/mL), and PBS respectively. OMVs induced S100A8 expression robustly in both BMDMs and MHS cells. S100A8 is a well-documented DAMP molecule [28]. To evaluate whether the induction of S100A8 is LPS- and TLR4-dependent, we again isolated BMDMs from TLR4 KO mice and treated the TLR4-null BMDMs with PBS, LPS, and OMVs. OMVs induce S100A8 in TLR4-null cells remarkably more than LPS induced S100A8, particularly at 25 µg/mL. Furthermore, a clear dose-response on S100A8 induction was observed in OMV-treated cells but not in LPS-treated cells (Figure 6D).

## 4. Discussion

Bacterial OMVs generally refer to the vesicles released from Gram-negative bacteria, despite the fact that Gram-positive bacteria also release vesicles [29]. OMVs transport bacterial cell signaling biochemicals which can function as PAMPs, including DNA, RNA, proteins, endotoxins, and virulence molecules [11]. The outer membrane of Gram-negative bacteria also consists of inner leaflet phospholipids and outer leaflet lipopolysaccharides (LPS) [30]. The OMV-carried endotoxic LPS has been widely documented in disease processes, and for many years, it has been hypothesized that LPS may play a major role in OMV-mediated macrophage activation. Despite being discovered by Drs. Chatterjee and J. Das a few decades ago [31], OMVs have not been comprehensively studied in terms of their significance and potential function in the pathogenesis of human diseases, particularly in the field of acute lung inflammation and injury. In the past decade, OMVs have been shown to mediate lung disease such as the hospital-acquired and ventilator-related pneumonia induced by OMVs derived from *A. baumannii, Pseudomonas,* and *E. coli* [16,32,33]. In these reports, it was suggested that OMVs function by activating TLR4/TLR2 -mediated pathways, presumably due to OMV-carried LPS. LPS-neutralizing peptides have also been found to inhibit OMV-induced activation of the inflammasome/IL-1 axis, suggesting an essential role of LPS in bacterial OMV-associated inflammatory responses [34]. On the other hand, emerging evidence suggests a multi-functional role of OMVs in the pathogenesis of human diseases, in addition to LPS. For example, OMVs contribute to innate host defense by absorbing antimicrobial peptides and bacteriophage [35], subsequently neutralizing environmental agents that target the outer membrane of Gram-negative bacteria [35].

Our report first explored and confirmed the functions of bacterial OMVs in the development of inflammatory lung responses after Gram-negative bacterial pneumonia. We next explored the underlying mechanisms. Interestingly, despite the fact that LPS has a significant impact, we found that OMVs likely exert their primary functions through LPS-independent pathways. We screened the TLR expressions in the presence and absence of OMVs. TLR1, TLR2, and TLR6 were robustly induced by OMVs, while TLR5 and TLR9 were significantly reduced, indicating that a multi-signaling pathway must play a role. Interestingly, TLR4, the most prominent receptor for LPS, remained unaffected after exposure to OMVs. To further explore the roles of TLRs in mediating OMV-induced macrophage activation and lung inflammation, we examined the effects of OMVs on TLR4-null cells. Our results showed that deletion of TLR4 robustly reduced the levels of LPS-induced pro-inflammatory cytokines but minimally affected the OMV-treated ones. These results indicate that OMVs exert their effects via multiple pathways in addition to TLR4 signaling. First, OMVs may facilitate the transfer of bacterial RNAs to the host cells [36]. The penetrated bacterial RNA in the eukaryotic cells may be detected by TLR3, TLR7, and TLR8 [37,38]. In our studies, despite the fact that TLR3, TLR4, TLR7, and TLR8 were not induced or suppressed by bacterial OMVs, they may still interact with OMV-cargos and activate signaling pathways. Furthermore, the activation of intracellular signaling pathways may be influenced by the number of OMVs which penetrate into the cells. OMVs potentially function as a repertoire of DAMPs/PAMPs. Presumably, the size of the OMVs may affect their penetration into cells, subsequently interfering with their intracellular signaling pathway activations. As previously reported, smaller OMVs have better penetration and interactions with Pattern Recognition Receptors (PRRs) [39,40]. Therefore, the results in our studies may not be extrapolated to other strains of bacteria. However, despite the fact that the signaling pathway may be different, the impacts of OMVs on innate immunity and host defense are similar.

OMVs exert multiple cellular functions on macrophages via multi-signaling pathways. Using qPCR array, we have also identified a variety of cytokines/chemokines which are strongly induced by OMVs, including, but not limited to CXCL1, 2, 9, 10, 13, and 16, CX3CL1, CCL5, 6, 21, 27, 28, and Chemerin. Furthermore, a variety of intra-cellular pro-inflammatory proteins, such as S100 protein A-8, were increased in macrophages after exposure to OMVs. Again, this effect is LPS-independent. Based on our observations, OMVs may be viewed as a repertoire of PAMPs/DAMPs which powerfully trigger numerous cell surface and intracellular receptors/signaling pathways, resulting in pro-inflammatory macrophage activation.

In the BALF obtained from mice treated with OMVs, a marked elevation of neutrophil infiltration was observed. This is consistent with the significant amounts of chemokines detected in OMV-treated macrophages. For example, CXCL belongs to the CXC chemokine family that acts as a chemoattractant for several immune cells, particularly neutrophils. In vivo, after exposure to Gram-negative bacteria, circulating and residing macrophage migrate to the local site of infection. Our study confirmed that OMV-treatment promotes macrophage and neutrophil infiltration and migration. Previous studies have indicated that the nature and severity of inflammatory responses caused by OMVs are variable, depending on the original bacteria which generate the OMVs [41]. As mentioned above, our studies confirmed that OMVs exert their effects via multiple signaling pathways. In addition to surface antigen interactions, such as TLRs, OMVs also interact with intracellular receptors and trigger intracellular signaling pathways. Therefore, the size and penetration of OMVs are presumably factors which contribute to the degree of macrophage activation and inflammatory responses. Different strains of Gram-negative bacteria may release OMVs with a different size, cargo, and amount of LPS, subsequently inducing different signaling pathways.

Another factor that may affect the functional roles of OMVs in macrophage activation and lung inflammation is the underlying mechanisms involved in OMV biogenesis. OMV biogenesis is a complex physiological process that is influenced by the strain of bacteria as well as many environmental factors, including the host conditions. Multiple different models of OMV biogenesis have been proposed [42]. For example, *A. baumanii* and *E. coli* release OMVs via reduced cross-linking between the outer membrane and the underlying peptidoglycan layer [43,44]. On the other hand, in the case of *Helicobacter pylori* [45] and *Shigella boydii* [46], the Tol-Pal system, which maintains bacterial membrane integrity, regulates their OMV production. Increased LPS and phospholipids of the outer membrane may alter the membrane curvature, resulting in OMV release [47]. Additionally, environmental changes potentially regulate OMV secretions, such as temperature. For example, the production of OMVs in *Serratia marcescens* [48] and *E. coli* [49] is altered by temperature changes. *Pseudomonas* produce more OMVs in the presence of antibiotics [50]. Although a highly conserved phospholipid transporter, VacJ/Yrb ABC, is proposed to regulate OMV generation in all Gram-negative bacteria, many biogenesis mechanisms of OMVs are species-specific. Thus, the functional roles of OMVs should be evaluated in a species-specific manner, with consideration of environmental factors too. The consequence of the complexity of OMV functions is that it adds more difficulties to the development of novel diagnostic and therapeutic strategies targeting bacterial OMVs. It is very possible that OMV-targeted diagnostic/therapeutic strategies must be tailored based on each group of OMVs. In addition to the limitations mentioned above, we studied *E. coli*-derived OMVs. *E. coli* does not commonly induce human pneumonia. However, although it is rarely considered to be a respiratory pathogen, it can cause severe respiratory disease. It has been reported that in non-cystic fibrosis patients, *E. coli*-associated, community-acquired pneumonia has a mortality rate of 11–21% and a disproportionally high rate of bacteremia [33,51]. Further studies should also explore the half-life and pharmacokinetics of OMVs in vivo.

In summary, our studies confirmed the pro-inflammatory activities of bacterial OMVs and revealed a complex, multi-signaling pathway which potentially mediates the functions of OMVs. Future development of diagnostic/therapeutic strategies may need to be based on particular OMVs which originate via specific mechanistic biogenesis and from different bacterial strains. Focusing on a single signaling pathway induced by OMVs may be futile.

## Figures and Tables

**Table 1:** List of primers used in qPCR.

Gene	Sequence (5′–3′)
TNFα-F	5′-GACGTGGAACTGGCAGAAGAG-3′
TNFα-R	5′-TTGGTGGTTTGTGAGTGTGAG-3′
IL1β-F	5′-GCAACTGTTCCTGAACTCAACT-3′
IL1β-R	5′-ATCTTTTGGGGTCCGTCAACT-3′
iNOS-F	5′-CAAGCTGAACTTGAGCGAGGA-3′
iNOS-R	5′-TTTACTCAGTGCCAGAAGCTGGA-3′
CXCL1-F	5′-CTGGGATTCACCTCAAGAACATC-3′
CXCL1-R	5′-CAGGGTCAAGGCAAGCCTC-3′
IL10-F	5′-GCTCTTACTGACTGGCATGAG-3′
IL10-R	5′_CGCAGCTCTAGGAGCATGTG-3′
TBP-F	5′-TCAAACCCAGAATTGTTCTCC-3′
TBP-R	5′-GGGGTAGATGTTTTCAAATGC-3′
S100a8-F	5′-CAAGGAAATCACCATGCCCTCTA-3′
S100a8-R	5′-ACCATCGCAAGGAACTCCTCGA-3′

**Table 2:** A summary of the upregulated chemokines with fold change >2 and the downregulated chemokine from the mouse chemokine array. OMV-treated MHS were compared to EXO-treated MHS and CXCL1 was the most up-regulated chemokine (*n* = 2).

Upregulated Chemokine	Fold
CXCL1	8.8238
CXCL2	5.5132
CXCL9	4.6757
CXCL10	4.6167
CX3CL1	3.0991
CCL27	2.8644
CCL6	2.8452
CCL5	2.6587
Chemerin	2.6376
CXCL16	2.4300
CCL28	2.3155
CCL21	2.3072
CXCL13	2.2632
**Downregulated Chemokine**	**Fold**
C5/C5a	0.35372

## Data Availability

The authors confirm that the data supporting the findings of this study are available within the article.
